# Oleic Acid‐Mediated Double Emulsification Enables Enhanced Aqueous Stability and Localized Photothermal Heating of Liquid Metal Droplets

**DOI:** 10.1002/smtd.71023

**Published:** 2026-09-09

**Authors:** Eunju Na, Youngwon Kim, Jihye Kim, Sang Kug Chung, Yongmin Cho, Bongseop Kwak, Joohyung Lee

**Affiliations:** ^1^ Department of Chemical Engineering Myongji University Yonginsi Republic of Korea; ^2^ College of Medicine Dongguk University Goyangsi Republic of Korea; ^3^ Department of Mechanical Engineering Myongji University Yonginsi Republic of Korea

**Keywords:** double emulsions, liquid metals, localized heating, photothermal therapy, water stability

## Abstract

Gallium‐based liquid metals (LMs) have emerged as promising photothermal agents owing to their broadband optical absorption, high photothermal conversion capability, and intrinsic fluidity. However, their practical implementation in aqueous and biological environments remains severely limited by water‐induced oxidation and hydrolysis, which progressively deteriorate their structural and photothermal stability. Here, we present a facile and potentially scalable double‐emulsion strategy that physically isolates LM nanodroplets from water without requiring complex surface modification. By dispersing eutectic gallium–indium (EGaIn) nanodroplets within an oleic acid (OA) intermediate phase and subsequently emulsifying the system in water, a yolk–shell‐type LM‐*in*‐OA‐*in*‐water (LM/OA/W) architecture is spontaneously formed through wettability‐driven confinement. The OA phase serves as both a barrier against water transport and a thermally insulating microenvironment. Consequently, the double‐emulsified LM exhibits improved aqueous stability and amplified photothermal performance compared with conventional LM/water dispersions, while retaining its morphology and functionality during prolonged aqueous storage and repeated near‐infrared irradiation. Furthermore, the double‐emulsion platform demonstrates low acute toxicity, effective photothermal suppression of three‐dimensional tumor spheroids, and facile incorporation of additional functional components. This work establishes wettability‐driven double emulsification as a simple yet versatile strategy for stabilizing and multifunctionalizing LM‐based photothermal systems.

## Graphical abstract

Oleic acid‐mediated double emulsification creates a yolk–shell‐like liquid metal photothermal platform in which the intermediate oil phase limits water transport to the liquid metal and reduces convection‐mediated heat dissipation from the irradiated region. This architecture enhances aqueous stability, promotes localized photothermal heating, and provides a modular framework for incorporating diverse functional materials.

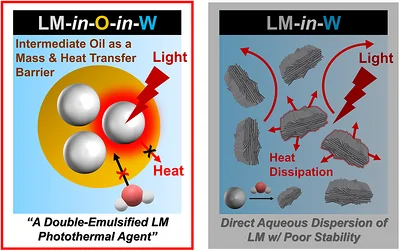

## 1 Introduction

Photothermal therapy (PTT) has emerged as a powerful and minimally invasive therapeutic modality that enables localized hyperthermia through light‐to‐heat conversion, offering high spatial precision and reduced systemic side effects [1, 2]. The effectiveness of PTT critically depends on the photothermal agents employed, which must exhibit strong broadband absorption, efficient photothermal conversion, and stability under physiological conditions.

Liquid metals (LMs), particularly gallium (Ga)‐based alloys, have recently attracted significant attention as next‐generation photothermal agents in the form of micro‐ or nano‐droplets, owing to their broadband optical absorption [3], high photothermal conversion efficiency [4, 5], and intrinsic fluidity that enables deformability and reconfigurability [6, 7]. These characteristics make LMs highly attractive for biomedical photothermal applications, including tumor ablation and antimicrobial treatments [8, 9].

Despite these advantages, the practical use of LMs in aqueous and biological environments remains severely limited by their poor water stability. Upon exposure to water, Ga‐based LMs readily undergo oxidation and hydrolysis, leading to the formation of crystalline hydroxides such as GaOOH, accompanied by hydrogen evolution [10, 11]. This process compromises their long‐term structural integrity and may ultimately degrade their photothermal performance.

To overcome this limitation, extensive efforts have been devoted to surface modification strategies, including polymer grafting [12, 13, 14, 15] and inorganic shell coating (Table S1) [4, 5, 11]. While effective to some extent, these approaches often involve complex synthetic procedures, harsh chemical reactions, or the use of potentially cytotoxic reagents, raising concerns regarding scalability, reproducibility, and biomedical safety. From a translational perspective, there is a clear need for a simpler and less invasive strategy that can stabilize LMs in aqueous environments.

Here, we propose a facile and potentially scalable double‐emulsion strategy that physically isolates LM nanodroplets from direct contact with water, eliminating the need for complex surface modification. By encapsulating LM nanodroplets within an intermediate oil phase, which preferentially associates with the LM surface, and subsequently dispersing them in an aqueous continuous phase, a robust LM‐in‐oil‐in‐water architecture is formed through a purely physical two‐step emulsification process (Scheme 1a). This configuration establishes an effective mass‐transfer barrier that reduces the water‐induced transformation of LM into crystalline hydroxides while simultaneously generating a thermally insulating microenvironment that promotes localized photothermal heating (Scheme 1b). Using eutectic Ga–In (EGaIn) and oleic acid (OA) as the model LM and reactive intermediate oil phase, respectively, we demonstrate that the double‐emulsion structure markedly improves the aqueous stability of LM nanodroplets while enhancing their photothermal performance. We also assess the acute aquatic toxicity of the resulting double‐emulsified LM using *Daphnia magna* (*D. magna*) and evaluate its therapeutic potential using a three‐dimensional (3D) multicellular tumor spheroid model. Finally, we show that the platform may readily accommodate additional functional components, such as therapeutic cargos or magnetically responsive nanoparticles for transport control. Together, these results establish the double‐emulsion strategy as a simple yet versatile platform for achieving both enhanced aqueous stability and multifunctionality in LM‐based photothermal therapeutic systems.

**SCHEME 1:**
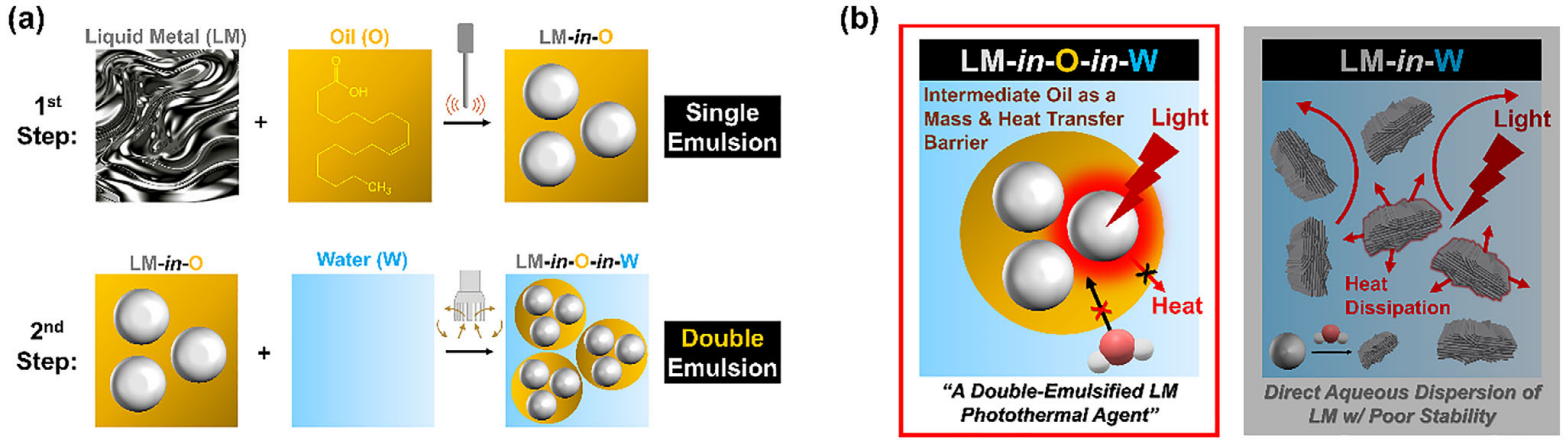
(a) A facile and potentially scalable double‐emulsification protocol for LMs. (b) A double‐emulsified LM photothermal agent proposed in this study, exhibiting enhanced aqueous stability and localized photothermal effects (left), compared with bare LM directly dispersed in water, which readily undergoes water‐mediated degradation and rapidly dissipates generated heat (right).

## 2 Results and Discussion

### 2.1 LM/OA Primary Emulsion

The first step in the preparation of the double emulsion is the formation of an LM‐*in*‐oil configuration, namely, an LM‐*in*‐oil single emulsion (see the first row of Scheme 1a), in which the LM and oil are EGaIn and OA, respectively. As shown in Figure 1a, the LM‐*in*‐OA (denoted as LM/OA) single emulsion was readily prepared by introducing bulk LM (2 vol%) into excess OA, followed by ultrasonication for approximately 10 min. As confirmed by dynamic light scattering (DLS) analysis, the sonication process readily produces submicrometer‐sized LM droplets (Figure 1b). Transmission electron microscopy (TEM) images further reveal that the resulting LM nanodroplets exhibit a spherical morphology (Figure 1c). Energy‐dispersive X‐ray spectroscopy (EDS) analysis of these particles shows the presence of Ga and In, the primary constituents of the LM, along with O and C signals. Notably, the C signal is particularly pronounced near the particle surface, indicative of the presence of adsorbed OA molecules.

**FIGURE 1:**
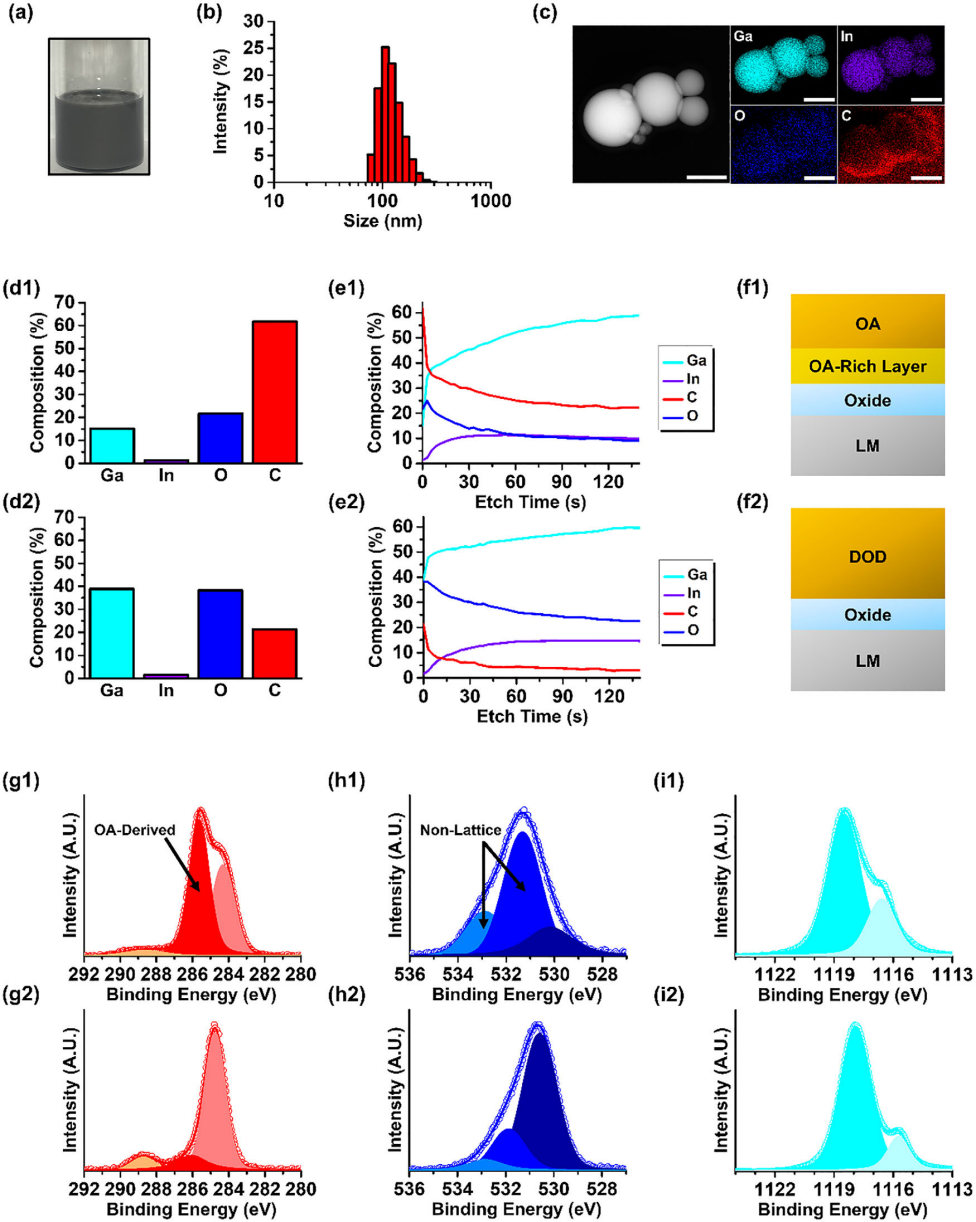
(a) Photograph of the LM/OA single emulsion. (b) Solvodynamic diameter distribution and (c) TEM/EDS analysis of LM nanodroplets dispersed in the LM/OA emulsion (scale bar: 250 nm). XPS elemental compositions at the outermost surfaces of LM nanodroplets dispersed in (d1) OA and (d2) DOD, together with depth profiles of their near‐surface elemental compositions obtained by Ar‐ion etching, as shown in (e1) and (e2), respectively. (f1, f2) Schematic representations of the proposed near‐surface structures of LM nanodroplets prepared in OA and DOD, respectively. High‐resolution XPS spectra of (g1, g2) C 1s, (h1, h2) O 1s, and (i1, i2) Ga 2p at the outermost surfaces of LM nanodroplets isolated from the LM/OA (g1, h1, i1) and LM/DOD (g2, h2, i2) single emulsions.

Even without an added stabilizer, LM droplets dispersed in OA remained colloidally stable for over one month (Figure S1), in marked contrast to the rapid sedimentation previously reported for LM droplets in pure nonpolar oils [16]. This stability likely arises because OA serves not only as the dispersion medium but also as an interfacially active species [14, 15] that preferentially associates with the LM‐droplet surface, thereby improving the compatibility of the LM droplets with the surrounding OA phase. To assess the retention of oil‐derived species at the LM surface, the LM/OA single emulsion was washed with excess hexane by centrifugation. The isolated LM droplets were then dried under ambient conditions for 24 h and analyzed by X‐ray photoelectron spectroscopy (XPS). As a nonpolar control oil lacking chemically reactive functional groups, dodecane (DOD) was used to prepare an LM‐*in*‐DOD (LM/DOD) single emulsion following the same procedure, and the LM droplets were isolated using an identical washing and drying protocol. We note that the surface chemistry of the dried, isolated LM droplets does not necessarily reproduce that of the droplets dispersed in the liquid oil phase. Nevertheless, because the OA‐ and DOD‐derived samples were subjected to identical ex situ handling, their relative differences provide comparative information regarding the retention of oil‐derived species at the LM surface. The surface elemental compositions obtained by XPS show that the LM/DOD sample contains relatively high proportions of Ga and O (Figure 1d2), as expected for a more exposed oxidized EGaIn surface, whereas the LM/OA sample exhibits a markedly higher C content (Figure 1d1). This pronounced carbon enrichment indicates that a substantial amount of OA‐derived carbonaceous material remained associated with the LM surface even after washing [17]. In the C 1s spectrum of the LM/OA sample, a low‐binding‐energy component is observed at approximately 284.3 eV (Figure 1g1), within the region generally associated with C–C/C–H and C = C carbon, whereas the LM/DOD spectrum is dominated by a hydrocarbon component centered at approximately 284.8 eV (Figure 1g2). The approximately 0.5 eV difference between these peak centers may reflect the contribution of olefinic carbon in OA and/or sample‐dependent differential charging. Notably, the LM/OA spectrum exhibits a pronounced component at approximately 285.6 eV. This feature lies approximately 1.3 eV above the low‐binding‐energy component within the same spectrum and occupies an intermediate‐binding‐energy region commonly associated with C–O‐containing carbon. A higher‐binding‐energy component near 288.6 eV is consistent with carboxyl/carboxylate carbon. Because intact OA contains no non‐carboxyl C–O carbon and is expected to be dominated by C–C/C–H environments, the unusually strong 285.6 eV component is not readily accounted for by intact OA alone under a simple chemical‐state assignment. We therefore provisionally attribute this feature to an OA‐associated intermediate‐binding‐energy carbonaceous population that includes oxygenated OA‐derived species, although differential charging of the heterogeneous organic/oxide/metal surface may also contribute to its apparent position and area. Consistent with this interpretation, the O 1s spectrum of the LM/OA sample exhibits substantially greater intensity in the approximately 531–533 eV region (Figure 1h1) than does the LM/DOD spectrum (Figure 1h2). These contributions are compatible with carboxyl/carboxylate oxygen, surface hydroxyl or oxyhydroxide species, and potentially additional oxygenated OA‐derived residues, supporting the presence of an oxygen‐rich OA‐derived surface environment. The lower relative contribution of the low‐binding‐energy O 1s component associated primarily with Ga–O environments may reflect attenuation by the carbon‐rich surface layer and/or modification of the near‐surface oxide environment by OA‐derived species. The Ga 2p spectrum of the LM/OA sample also exhibits an apparent displacement toward higher binding energy (Figure 1i1) relative to that of the LM/DOD sample (Figure 1i2). Given the heterogeneous electrical character of the organic/oxide/metal structure and the unreferenced binding‐energy scale, this difference is interpreted as evidence of an altered near‐surface chemical and/or electrostatic environment of Ga. Depth‐profiling analysis additionally reveals substantially higher near‐surface carbon contents for the LM/OA sample (Figure 1e1) than for the LM/DOD sample (Figure 1e2). Taken together, although the ex situ XPS data do not establish a unique interfacial bonding structure, they consistently indicate that a substantially greater amount of OA‐derived carbonaceous material remains associated with the LM surface even after washing. This strong retention of OA‐derived species may improve the compatibility between the LM droplets and the surrounding OA phase (Figure 1f1), in contrast to the limited interfacial association observed in the DOD medium (Figure 1f2), thereby contributing to the long‐term colloidal stability of the primary LM/OA emulsion (Figure S1). This colloidal stability, in turn, facilitates reliable processing during the subsequent double‐emulsification step.

### 2.2 Wettability‐Driven Configuration of LM/OA/W Double Emulsion

To construct the LM/OA/W double emulsion, the primary LM/OA emulsion was further dispersed in an equal volume of an aqueous poly(vinyl alcohol) (PVA) solution. PVA was employed as a water‐soluble stabilizer for the OA droplets dispersed in the continuous aqueous phase.

Before discussing the OA‐based system, it is useful to consider the wetting behavior of the native oxide‐skinned LM surface at a water–nonpolar‐oil interface. Previous studies have shown that such LM surfaces can be wetted by both the aqueous and nonpolar oil phases [11, 18]. We further confirmed this behavior by placing a water (W) droplet on an LM droplet immersed in DOD (Figure 2a1 and Movie S1). Rather than rolling off, the water droplet remained attached to the LM surface and exhibited an apparent three‐phase contact angle of approximately 70°, consistent with previous reports [16, 19]. This water–oil “amphiphilicity” enables LM microdroplets to function as interfacial stabilizers. When a small quantity of LM microdroplets is introduced into and mixed with a water–oil system, a Pickering‐type [20] emulsion can readily form, with the LM droplets adsorbing at the oil–water interface and stabilizing the dispersed oil droplets (Figure 2c1) [11, 21]. Here, the term “Pickering‐type” does not refer to systems in which LM droplets constitute the dispersed phase and are stabilized by external solid particles, such as cellulose nanocrystals [22, 23]. Rather, it refers to a distinct configuration in which the LM droplets themselves adsorb at the oil–water interface and stabilize the dispersed oil droplets, thereby functioning as droplet‐type “mutant” Pickering emulsifiers [11]. Figure 2b1 shows an optical micrograph of the resulting DOD‐*in*‐water (DOD/W) emulsion, prepared by first dispersing LM in DOD and subsequently mixing the primary LM/DOD emulsion with deionized water. The large oil droplets appeared dark because their surfaces were densely covered with optically dark LM droplets, consistent with previous observations [11]. In this configuration, however, the interfacially adsorbed LM droplets remain directly exposed to the aqueous phase, rendering them susceptible to water‐induced transformation into crystalline hydroxide products during prolonged storage [11].

**FIGURE 2:**
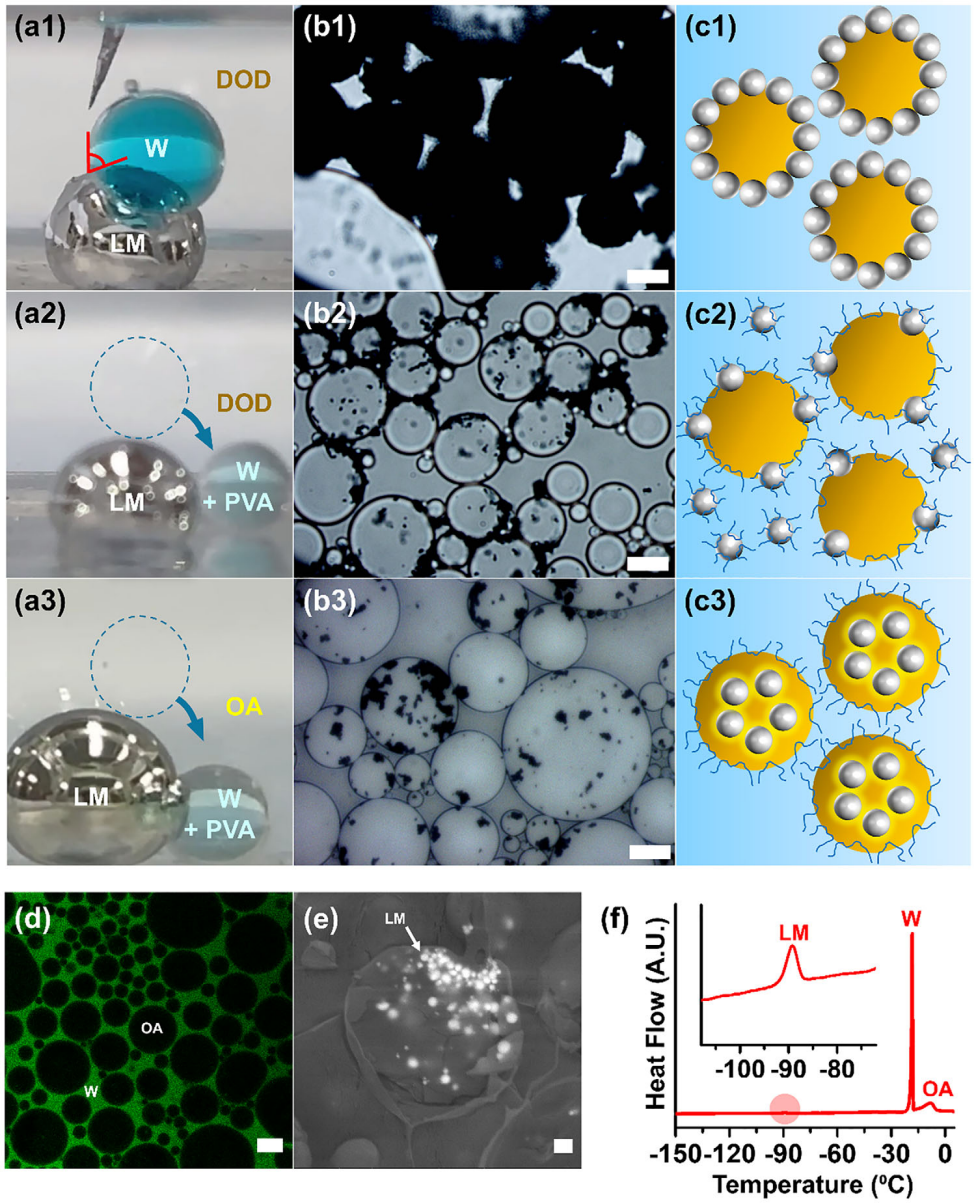
Observation of the wetting configuration among the three phases: (a1) LM (droplet), W (droplet), and DOD (bulk); (a2) LM (droplet), W containing dissolved PVA (droplet), and DOD (bulk); and (a3) LM (droplet), W containing dissolved PVA (droplet), and OA (bulk). Optical micrographs of the corresponding emulsions: (b1) LM/DOD/W without PVA, (b2) LM/DOD/W with PVA, and (b3) LM/OA/W with PVA. Schematics of the corresponding emulsions: (c1) LM/DOD/W without PVA, (c2) LM/DOD/W with PVA, and (c3) LM/OA/W with PVA. (d) CLSM, (e) cryo‐SEM, and (f) DSC analysis of the LM/OA/W emulsion containing PVA. The scale bars in (b) and (d) represent 10 µm, while the scale bar in (e) represents 1 µm. The inset in (f) shows an enlarged view of the data near −90°C. For all emulsion samples, the oil‐to‐water ratio was fixed at 5:5, and the LM content was fixed at 1 vol% of the total volume. In all PVA‐containing samples, 1 wt% PVA was dissolved in the water phase.

Upon the addition of PVA to the aqueous continuous phase, the surface‐active polymer preferentially adsorbs at the nonpolar oil–water interface [24]. Consistent with this interfacial activity, droplets of the aqueous PVA solution introduced into the DOD phase no longer adhered to or appreciably wetted the LM surface (Figure 2a2 and Movie S2), presumably because PVA rapidly adsorbed at the newly formed water–DOD interface. During secondary emulsification, rapid PVA adsorption at the DOD–water interfaces likewise suppressed the interfacial adsorption of LM droplets (Figure 2b2). Under these conditions, LM droplets displaced from the transient PVA‐stabilized water–oil interface were not retained within the dispersed DOD phase but instead redistributed into the aqueous continuous phase. This behavior is likely facilitated by the adsorption of PVA onto the LM surface, which increases its hydrophilicity and promotes aqueous dispersibility [25]. Consequently, dispersed DOD droplets and independently dispersed LM droplets coexisted in the aqueous continuous phase, with only a small fraction of LM microdroplets remaining weakly attached to the surface of the larger oil droplets (Figure 2c2). Although PVA adsorption may impart some colloidal stabilization of LM droplets in water, it does not eliminate their intrinsic susceptibility to water‐induced chemical transformation [11].

In contrast, the OA‐based system exhibited a markedly different wetting behavior. Macroscopic water droplets placed on an LM droplet immersed in OA did not remain attached to the LM surface but instead readily rolled off (Figure 2a3 and Movie S3). Notably, this behavior was observed both in the presence and absence of PVA in the aqueous phase (Figure S2 and Movie S4), in sharp contrast to the nonpolar DOD‐based system. This reproducible roll‐off behavior indicates that the OA‐conditioned LM surface has substantially lower affinity for the aqueous phase. Corresponding behavior was observed during secondary emulsification. When the primary LM/OA emulsion was homogenized with an aqueous PVA solution, LM droplets displaced from the transient OA–water interfaces did not migrate appreciably into the aqueous continuous phase. Instead, they remained predominantly confined within the dispersed OA droplets (Figure 2b3). Thus, both the macroscopic wetting experiment and the microscopic emulsion configuration indicate that the OA‐conditioned LM surface preferentially remains associated with the OA phase rather than the aqueous phase. The wetting observations alone do not identify the underlying interfacial chemistry. However, the comparative ex situ XPS analysis discussed above provides complementary evidence for the persistent association of OA‐derived species with the LM surface. Despite identical washing and drying procedures, LM isolated from OA retained a substantially greater amount of near‐surface carbonaceous material than LM isolated from DOD. Although the ex situ spectra do not directly reproduce the liquid‐state interface, they clearly indicate stronger retention of OA‐derived species at the LM surface. Taken together, the wetting behavior, the resulting emulsion configuration, and the comparative XPS results support a model in which a persistent OA‐rich interfacial environment reduces water adhesion and promotes retention of LM droplets within the OA phase.

Accordingly, pre‐dispersing LM in OA, rather than in a nonpolar oil such as DOD, directs the LM droplets to remain within the dispersed OA phase during subsequent emulsification in water. This process yields a multicore, yolk–shell‐like LM‐*in*‐OA‐*in*‐water (LM/OA/W) double‐emulsion architecture (Figure 2c3) without requiring additional surface functionalization or microfluidic processing. Because batch‐type rotor–stator homogenization does not produce monodisperse OA droplets or prescribe the number of LM droplets encapsulated within each droplet, both the OA‐droplet size and the internal LM loading vary among individual droplets. Nevertheless, optical microscopy analysis of multiple aliquots collected from several independently prepared batches showed that LM‐containing OA droplets with comparable size distributions were reproducibly generated (Figure S3). The optically identifiable double‐emulsion droplets had a mean diameter of 11.94 ± 6.65 µm. The secondary emulsification process also produced numerous OA droplets only a few micrometers in diameter, as expected for batch homogenization. However, because the presence of nanoscale LM droplets within these smaller OA droplets could not be reliably resolved by optical microscopy, these droplets were excluded from the size‐distribution analysis. Accordingly, the reported size distribution represents OA droplets containing optically visible LM rather than the entire OA‐droplet population. To further quantify the preferential confinement of LM within the OA phase, an apparent two‐dimensional confinement fraction, *F*_conf,2D_, was determined from multiple high‐magnification optical micrographs (Figure S4). This parameter was defined as the projected area of LM located within the OA droplets divided by the total projected LM area (see the Supporting Information for a detailed description of the calculation). The mean *F*_conf,2D_ values above 99% from multiple analyzed micrographs indicate that most of the optically detected LM area was confined within the dispersed OA droplets immediately after preparation. Confocal laser scanning microscopy (CLSM) analysis of the LM/OA/W system confirmed that the oil phase formed discrete dispersed droplets within the continuous aqueous phase, with no detectable aqueous‐phase signal inside the oil droplets under the present imaging conditions (Figure 2d). Furthermore, cryogenic scanning electron microscopy (cryo‐SEM) analysis of an isolated oil droplet in backscattered electron (BSE) mode revealed that the encapsulated LM nanodroplets retained a spherical morphology without noticeable deformation (Figure 2e). Additional images are provided in Figure S5 to demonstrate the reproducibility of this observation. Differential scanning calorimetry (DSC) analysis performed during cooling of the LM/OA/W system showed distinct exothermic peaks attributable to the liquid‐to‐solid phase transitions of the individual constituents (Figure 2f). In particular, the LM crystallization peak observed at approximately −89°C, which was absent from the LM‐free OA/W emulsion (Figure S6), was consistent with previously reported values for nanosized EGaIn droplets [26]. Together, these observations indicate that the LM nanodroplets retain their spherical morphology and characteristic strongly supercooled crystallization behavior after the double‐emulsification process. Similar LM‐*in*‐oil‐*in*‐water configurations were also obtained using other oils containing polar functional groups, including octanoic acid and canola oil (Figure S7). These observations suggest that the confinement behavior is not unique to OA; however, further systematic studies using a broader range of reactive oils with different chemical functionalities and physicochemical properties will be required to establish the general applicability of this method.

### 2.3 Intermediate Oil Phase as a Barrier to Water Transport and Heat Dissipation

#### 2.3.1 Enhanced Aqueous Stability

Figure 3a compares the time‐dependent evolution of the three emulsions shown in Figure 2b during storage under ambient conditions. The LM/DOD/W emulsion prepared without PVA was initially macroscopically homogeneous; however, large internal voids progressively developed, and the sample eventually transformed into a fragile, crumbly structure that readily disintegrated upon slight agitation (Figure 3a1). Similar instability was observed in the PVA‐containing LM/DOD/W system, in which the dispersed phase gradually rose to the top of the storage vial (Figure 3a2). Morphological analysis of particles collected after approximately 1 week from an independently prepared PVA‐containing LM/DOD/W emulsion revealed stacked, anisotropic aggregates rather than the original spherical LM droplets (Figure 3c1). XRD analysis identified these structures as crystalline GaOOH (Figure 3d1), which can form through the reaction between LM and water (e.g., 2Ga + 4H_2_O → 2GaOOH + 3H_2_↑) [10]. These observations indicate that hydrogen evolution resulting from the LM–water reaction likely caused structural disruption and flotation of the dispersed phase, while PVA adsorption alone was insufficient to prevent the water‐induced transformation of LM when droplets were dispersed in the aqueous continuous phase.

**FIGURE 3:**
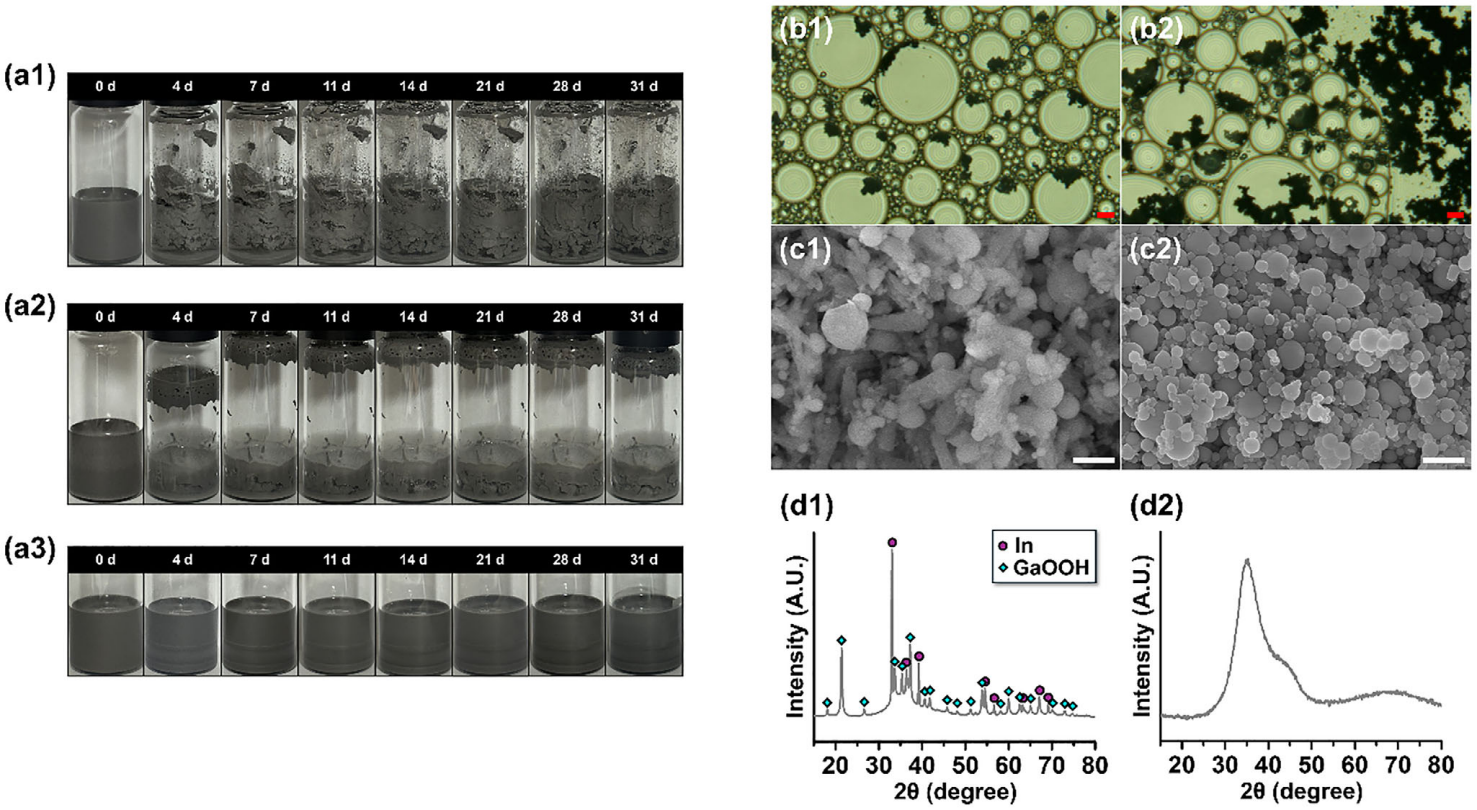
Photographs showing the time‐dependent evolution of three emulsion samples over 31 days after preparation: (a1) LM/DOD/W without PVA, (a2) LM/DOD/W with PVA, and (a3) LM/OA/W with PVA. Optical micrographs of the samples collected from the (b1) top and (b2) bottom layers of the 31‐day‐aged PVA‐containing LM/OA/W emulsion (scale bar: 10 µm). SEM images of particles collected from independently prepared emulsion samples aged for 7 days: (c1) LM/DOD/W with PVA and (c2) LM/OA/W with PVA (scale bars: 1 µm). (d1, d2) Corresponding XRD patterns of the particles shown in (c1) and (c2), respectively. For all samples, the oil‐to‐water volume ratio was fixed at 5:5, and the LM content was fixed at 1 vol% of the total volume. The aqueous phase of all PVA‐containing samples contained 1 wt% PVA.

In contrast, the LM/OA/W double emulsion exhibited markedly enhanced stability. The overall sample height remained essentially unchanged over 1 month, and no visible signs of bubble formation or gross structural disruption were observed (Figure 3a3). LM droplets recovered from an independently prepared sample after approximately one week of aging largely retained their initial spherical morphology (Figure 3c2). Consistently, XRD analysis showed no discernible reflections attributable to crystalline GaOOH and instead displayed only broad diffuse scattering (Figure 3d2) [27]. These results indicate that the water‐induced transformation of LM into crystalline hydroxide products was substantially reduced in the LM/OA/W system, supporting the role of the encapsulating OA phase as a mass‐transfer barrier that limits water access to the LM droplets. These findings do not imply that corrosion processes, including partial oxidation of LM, were completely prevented. The atomic‐scale stability of individual LM droplets over substantially longer periods will require further evaluation using more quantitative and surface‐sensitive measurements. Nevertheless, the present formulation‐level stability study demonstrates that the OA‐based formulation behaves distinctly from the nonreactive DOD‐based control in limiting the water‐induced formation of crystalline hydroxides, gas‐bubble generation, and the resulting emulsion destabilization. Although various strategies have previously been explored to improve the aqueous stability of LM (Table S1), the present method provides a relatively simple approach, with its enhanced stability supported by mutually consistent macroscopic observations, microscopy, and XRD analysis. The formulation also maintained its macroscopic emulsion stability, and the microscopic double‐emulsion configuration remained intact in phosphate‐buffered saline (PBS) at 37°C throughout the one‐week test period. Moreover, the recovered LM droplets retained their spherical morphology without developing crystalline hydroxide structures (Figure S8).

Although the overall emulsion height remained unchanged, the aged LM/OA/W sample gradually developed vertical stratification into upper and lower layers. Optical microscopy showed that the upper layer consisted primarily of relatively LM‐lean OA droplets (Figure 3b1), whereas the lower layer contained denser, LM‐rich OA droplets together with a visible population of LM particles located outside the OA droplets (Figure 3b2). Such independently dispersed LM particles were rarely observed immediately after preparation and may, at least in part, have originated from gradual release during aging, possibly because of changes in the LM–OA interfacial state or transient desorption of OA‐derived species. However, some of the apparently free LM particles may also have been generated through mechanical rupture of heavily loaded OA droplets during sampling and microscopic specimen preparation. Accordingly, the local abundance of free LM observed in an individual micrograph cannot be directly interpreted as their fraction in the bulk formulation. Despite the presence of these apparently free particles, no appreciable gas‐bubble formation or gross destabilization of the emulsion was observed over 31 days. Thus, their presence did not measurably compromise the macroscopic stability of the overall formulation during the observation period. Notably, the lower layer was not dominated by independently sedimented LM particles but also contained numerous intact OA droplets with high internal LM loadings. If free OA droplets and free LM particles had separated independently, the relatively low‐density OA droplets would have creamed toward the top, whereas the denser LM particles would have sedimented. The accumulation of intact LM‐rich OA droplets in the lower layer is therefore consistent with an increase in their effective density caused by the encapsulated LM. Furthermore, the stratified droplets could be readily redispersed throughout the aqueous continuous phase by gentle shaking, indicating that the stratification arose primarily from reversible, density‐driven separation rather than irreversible coalescence or complete emulsion breakdown.

The LM/OA/W double‐emulsion structure was retained even when the OA content was reduced to an OA:W volume ratio of 1:9 (Figure S9a). At a fixed overall LM content (1 vol%), decreasing the OA fraction increased the LM loading within individual OA droplets (Figure S9b). In particular, for the double emulsion prepared at the lowest OA content (OA:W = 1:9), many of the denser, LM‐rich OA droplets collected from the bottom layer were nearly filled with LM particles (Figure S10a). Importantly, despite the reduced OA content, the LM droplets remained predominantly confined within the OA phase and were largely excluded from the OA–water interface. After 7 days of aging, the recovered LM droplets retained their spherical morphology and did not develop the stacked, anisotropic structures associated with the crystalline hydroxide products observed in the DOD‐based control (Figure S10b). These observations indicate that the reduced water‐induced structural transformation of the LM droplets was maintained even at a relatively low OA fraction, further supporting the proposed barrier function of the intermediate OA phase.

#### 2.3.2 Localized Photothermal Heating

To evaluate the photothermal performance of the double‐emulsified LM, an 808 nm near‐infrared (NIR) laser—commonly used in LM‐based therapeutic applications [4, 5, 6, 7, 8, 9]—was irradiated onto the top layer of the LM/OA/W sample (Figure 4a). We note that the present double‐emulsified photothermal system is structurally distinct from previously reported Pickering‐type systems [11, 28, 29, 30] in which photothermal agents are attached to the outer surfaces of emulsified oil droplets. In the present formulation, the LM photothermal agents are instead embedded within the dispersed OA droplets. As shown in Figures 4a and S11b, the temperature at the irradiation spot in the LM/OA/W sample rapidly increased by ≈59°C within 10 s, significantly exceeding that of the LM/W single emulsion, in which LM is directly dispersed in the aqueous continuous phase without an intermediate oil layer (ΔT ≈ 18°C). Because the two formulations contained the same initial LM loading and were evaluated under identical irradiation conditions, this comparison reflects their overall formulation‐level photothermal performance. Several factors may contribute to the substantially greater temperature rise of the double‐emulsified formulation. First, LM droplets directly dispersed in water exhibit pronounced physical and chemical instability. After only 10 s of NIR irradiation, the sedimentation layer at the bottom of the LM/W sample visibly increased in thickness (Figure 4b), indicating rapid colloidal destabilization and depletion of photothermally active LM from the upper irradiation region. Localized heating may further accelerate particle aggregation and sedimentation [11], as well as the water‐induced transformation of LM into crystalline hydroxide products [10]. During the second daily heating–cooling cycle, performed on Day 2, sedimentation became more pronounced, accompanied by increased optical transparency near the irradiation region (see the column labeled *2 d* in Figure 4b). Because depletion of LM from the irradiation region was already sufficiently severe to compromise the reliability of the temperature measurement, the corresponding temperature‐evolution profile is not presented. The material accumulated at the bottom also developed a loosely packed, porous appearance, likely associated with gas‐bubble evolution during the LM–water reaction.

**FIGURE 4:**
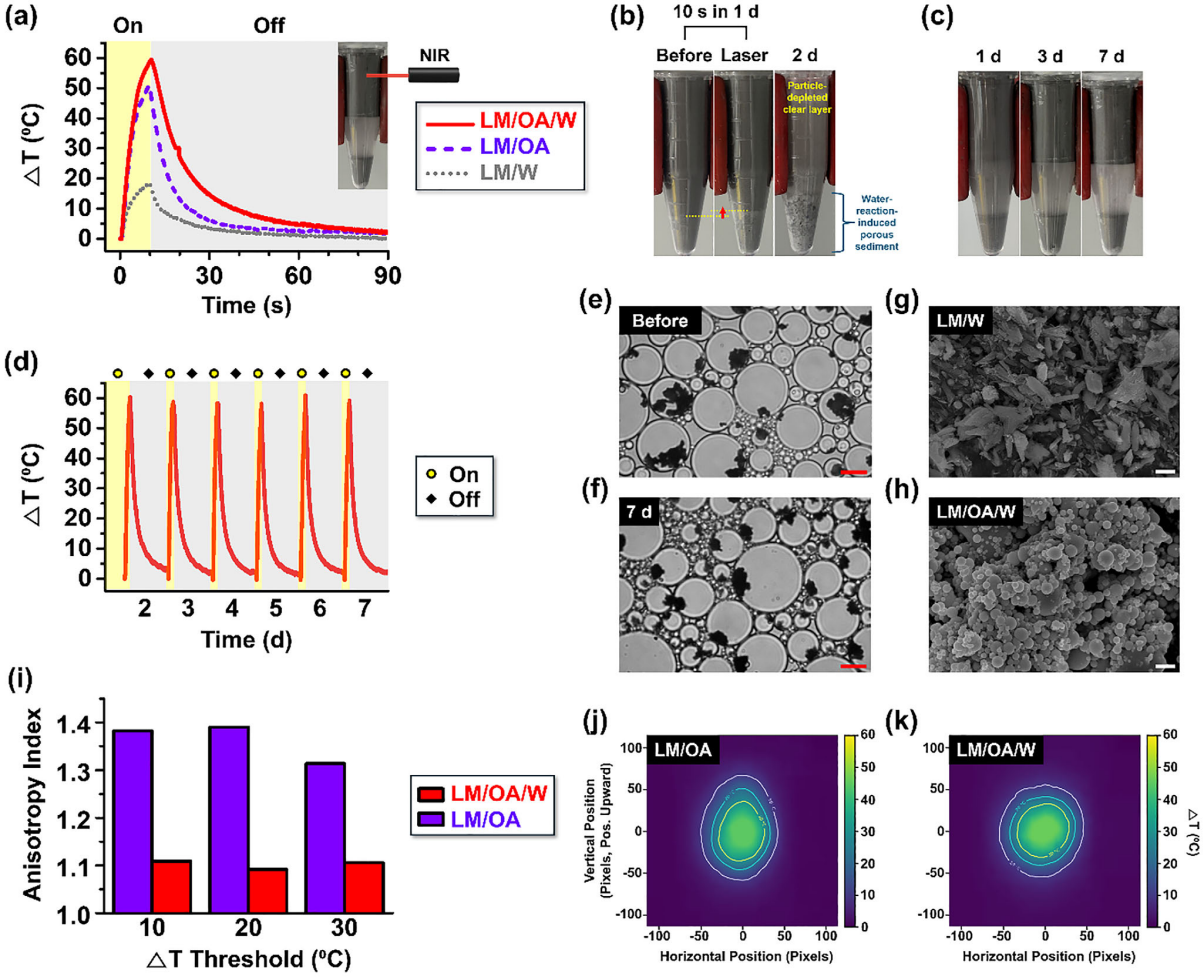
(a) Temperature‐rise (ΔT) profiles of various LM emulsion formulations during 10 s of NIR irradiation and subsequent natural cooling (yellow‐shaded region: NIR on; gray‐shaded region: NIR off): LM/OA/W double emulsion (OA:W volume ratio = 5:5), LM/OA single emulsion, and LM/W single emulsion. The inset illustrates the sample geometry and the location of NIR irradiation. The LM loading was fixed at 1 vol% in all formulations, and the aqueous phases of the LM/OA/W and LM/W formulations contained 1 wt% PVA. Photographs showing the macroscopic appearances of (b) LM/W before and after the first 10 s of irradiation and on Day 2, and (c) LM/OA/W on Days 1, 3, and 7 during the daily photothermal heating–cooling experiment. (d) Temperature‐rise profiles of LM/OA/W over seven daily photothermal heating–cooling cycles, demonstrating the repeatability of its photothermal response. Optical micrographs of the LM/OA/W emulsion (e) before irradiation and (f) after seven daily heating–cooling cycles (scale bars: 10 µm). SEM images of materials recovered from (g) LM/W and (h) LM/OA/W after daily 10 s NIR irradiation for 7 days (scale bars: 1 µm). (i) Anisotropy indices of the thermal‐footprint contours at ΔT thresholds of 10°C, 20°C, and 30°C for LM/OA and LM/OA/W. Pixel‐resolved ΔT maps and corresponding thermal‐footprint contours at the end of the 10 s NIR irradiation period for (j) LM/OA and (k) LM/OA/W.

In contrast, the LM/OA/W formulation exhibited no noticeable changes in its macroscopic appearance (Figure 4c) or OA‐droplet size (Figure 4e,f) over seven daily irradiation cycles. SEM analysis of the materials recovered after the 7‐day irradiation experiment further revealed marked differences between the two formulations. The material recovered from the LM/W sample predominantly exhibited anisotropic morphologies characteristic of crystalline transformation products (Figure 4g), whereas LM recovered from the double‐emulsified formulation largely retained its spherical morphology (Figure 4h). These results indicate that the encapsulating OA phase helps preserve the structural integrity of LM under both ambient aqueous conditions and repeated NIR irradiation. No systematic deterioration in the photothermal response of the LM/OA/W formulation was also observed over the seven daily heating–cooling cycles (Figure 4d). This durable response is consistent not only with preservation of the LM morphology but also with the spatial retention of photothermally active LM within the upper irradiation region. In the LM/W dispersion, freely suspended LM droplets can undergo aggregation, gravitational settling, and redistribution driven by thermally induced fluid motion, progressively depleting the local LM concentration near the irradiation spot. In the LM/OA/W formulation, by contrast, the small and dense LM droplets are confined within larger OA droplets and therefore cannot independently migrate or sediment through the aqueous phase. This compartmentalization limits the redistribution and local depletion of the LM phase, helping maintain a relatively stable concentration of photothermally active LM near the irradiation region. The combined preservation of LM morphology and spatial distribution therefore contributes to the greater and more durable localized temperature rise observed for LM/OA/W compared with the direct LM/W dispersion.

To further investigate the spatial characteristics of photothermal heating in the double‐emulsified system, the time‐dependent temperature evolution around the irradiation region and the spatial temperature distribution at the end of the 10 s irradiation period were analyzed by IR thermography (Movie S6, Figure S11b, and Figure 4k). As the non‐compartmentalized control in which LM droplets remain freely dispersed within a single continuous phase, the LM/OA single emulsion was selected instead of LM/W (Movie S5, Figure S11a, and Figure 4j). In the OA environment, water‐induced chemical transformation of LM is avoided, while the strong compatibility between OA and the LM surface provides relatively high colloidal stability with limited sedimentation over the experimental period (Figure S12).

Pixel‐resolved temperature maps were used to delineate connected thermal‐footprint regions at ΔT thresholds of 10°C, 20°C, and 30°C. The temperature fields observed for LM/OA/W (Figure 4k) were consistently more isotropic than those observed for LM/OA (Figure 4j). To quantify this difference, the connected thermal footprint containing the laser irradiation center at each ΔT threshold was identified and fitted with a second‐moment equivalent ellipse. The anisotropy index was then calculated as the ratio of the major to minor axis lengths of the fitted ellipse (Figure 4i; a schematic illustration of the analysis procedure is provided in Figure S11c). An anisotropy index of 1 represents a circular temperature distribution, whereas larger values indicate increasing anisotropy. At all three ΔT thresholds, LM/OA exhibited higher anisotropy indices than LM/OA/W, consistent with the more elongated thermal footprints observed for LM/OA. This anisotropy is consistent with buoyancy‐driven natural convection, in which the locally heated fluid becomes less dense and rises, carrying heat upward and away from the irradiation region. In contrast, the LM/OA/W formulation exhibited less vertically elongated and more circular thermal footprints, with the anisotropy indices closer to unity. This reduced anisotropy indicates diminished directional convective transport. Because the OA phase in the double‐emulsion configuration is partitioned into discrete droplets rather than forming a continuous bulk phase, the development of a coherent buoyancy‐driven flow within the heated oil phase is restricted, thereby reducing the large‐scale upward redistribution of heat. We note that the absolute temperature difference between LM/OA and LM/OA/W cannot be used to quantitatively isolate the contribution of the double‐emulsion architecture, because the two formulations may differ in optical scattering, interfacial light transmission, and the spatial distribution of LM within the irradiated region. Accordingly, the comparison is intended to evaluate their formulation‐level spatial heating behavior rather than to extract an architecture‐specific enhancement in photothermal conversion. Nevertheless, because the samples were examined under the same irradiation geometry, the calculated anisotropy indices provide a useful dimensionless measure of directional heat spreading. In this context, localized heating refers to reduced convection‐mediated heat transport away from the irradiation region, rather than necessarily to a smaller absolute isotherm area. Because LM/OA/W reaches a higher peak temperature, it may exhibit a comparable or even larger thermal‐footprint area at a given ΔT threshold. Its lower anisotropy nevertheless indicates reduced upward convective spreading and greater retention of heat around the irradiation region. Finally, after cessation of NIR irradiation, the LM/OA/W formulation cooled more slowly than the LM/OA single emulsion (see the laser‐off region in Figure 4a). Together with the reduced thermal anisotropy, this slower cooling behavior is consistent with slower heat dissipation and enhanced local heat retention in the compartmentalized double‐emulsion system.

### 2.4 Preliminary Biological Evaluation and Multifunctional Potential of Double‐Emulsified LM

#### 2.4.1 Acute Aquatic Toxicity Assessment Using *D. magna*

To evaluate the acute toxicity of the double‐emulsified LM at the whole‐organism level, we performed in vivo exposure assays using *D. magna*. *D. magna* is a standard aquatic test organism used in the OECD Test Guideline (TG) 202 acute immobilization assay [31], and has also been widely employed to assess sublethal physiological and behavioral responses to chemical stressors [32].

Microscopic examination revealed clear differences in the distribution of ingested LM within the digestive tract. In control organisms not exposed to LM, no dark deposits were observed in the gut region (Figure 5a1). In contrast, LM‐exposed *D. magna* exhibited visible accumulation of LM deposits within the digestive tract, confirming oral uptake of the water‐ dispersed LM formulations. In the case of the LM/W single emulsion (Figure 5a2), ingested LM droplets appeared as densely aggregated deposits within the gut. By comparison, for the LM/OA/W double emulsion (Figure 5a3), gut‐associated LM droplets were more diffusely distributed, suggesting that the dispersed state of LM droplets conferred by double emulsification was at least partially preserved within the gastrointestinal environment.

**FIGURE 5:**
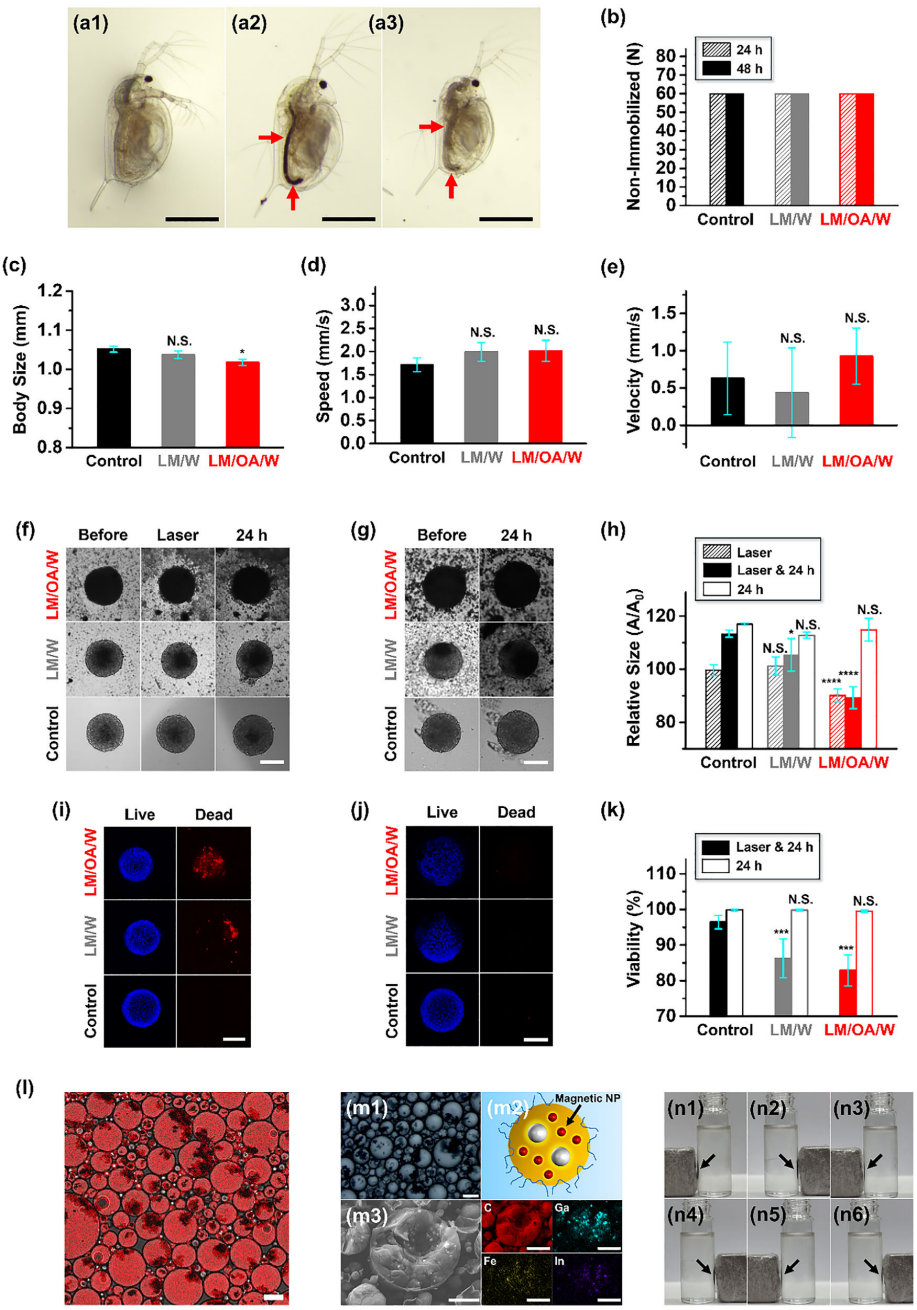
Preliminary biological assessment and multifunctionality of LM/OA/W double emulsions. (a1–a3) Representative optical micrographs of *D. magna* after 48 h exposure to diluted formulations: (a1) control without emulsion exposure, (a2) LM/W, and (a3) LM/OA/W. Red arrows indicate LM‐containing material observed within the digestive tract (scale bars: 0.5 mm). (b) Numbers of non‐immobilized *D. magna* individuals among 60 individuals per group after 24 and 48 h exposure. (c) Body size, (d) spontaneous swimming speed, and (e) phototaxis‐associated vertical swimming velocity after 48 h exposure (mean ± SE, *n* ≥ 30 per group). Positive and negative velocity values indicate movement toward and away from the light source, respectively. (f) Representative bright‐field images of BT‐474 multicellular tumor spheroids treated with LM/OA/W (top row), LM/W (middle row), or no emulsion (denoted as *Control*; bottom row), acquired after 24 h preincubation and immediately before NIR irradiation (denoted as *Before*; left column), immediately after 10 min NIR irradiation (denoted as *Laser*; middle column), and after a further 24 h incubation (denoted as *24 h*; right column) (scale bar: 200 µm). (g) Corresponding bright‐field images of nonirradiated spheroids acquired after the same 24 h preincubation (denoted as *Before*; left column) and after a further 24 h incubation without NIR irradiation (denoted as *24 h*; right column) (scale bar: 200 µm). (h) Relative spheroid area (A/A_0_) immediately after 10 min NIR irradiation (denoted as *Laser*; hatched bars), after a subsequent 24 h incubation (denoted as *Laser & 24 h*; filled bars), and after a corresponding 24 h incubation without NIR irradiation (denoted as *24 h*; open bars) (mean ± SD, *n* = 4 per group). A_0_ denotes the spheroid area measured after the initial 24 h preincubation. (i) CLSM images of irradiated spheroids after the subsequent 24 h incubation, showing Hoechst 33342‐stained nuclei (blue; denoted as *Live*; left column) and propidium iodide‐positive membrane‐compromised cells (red; denoted as *Dead*; right column) (scale bar: 200 µm). (j) Corresponding CLSM images of nonirradiated spheroids after the subsequent 24 h incubation (scale bar: 200 µm). (k) Cell viability measured after dissociation of spheroids following the 24 h postirradiation incubation (denoted as *Laser & 24 h*; filled bars) or the corresponding nonirradiated incubation (*24 h*; open bars) (mean ± SD, *n* = 4 per group). For (c–e), each emulsion‐treated group was compared with the blank control group. For (h) and (k), each LM‐containing formulation was compared with the corresponding blank control under the same irradiation and incubation conditions. Statistical significance was evaluated using the Kruskal–Wallis test followed by Dunn's post hoc test with Bonferroni correction (*adjusted *p* < 0.05, **adjusted *p* < 0.01, ***adjusted *p* < 0.001, and ****adjusted *p* < 0.0001; N.S., not significant). (l) CLSM image of LM/OA/W containing Nile Red in the OA phase (scale bar: 10 µm). Magnetically responsive LM/OA/W containing Fe_3_O_4_ nanoparticles co‐dispersed in the OA phase: (m1) optical micrograph, (m2) schematic illustration, and (m3) cryo‐SEM image and corresponding EDS elemental maps of the OA droplets (scale bars: 10 µm). (n1–n6) Sequential photographs showing magnetic manipulation of double‐emulsified LM within the continuous aqueous phase using a neodymium magnet.

In the 48‐h acute immobilization assay conducted in accordance with OECD TG 202, no immobilization was observed in any treatment group at either 24 or 48 h (Figure 5b), indicating no detectable acute immobilization toxicity under the tested conditions. Over the same exposure period, a slight reduction in average body size (within ∼5%) was observed in the groups treated with double‐emulsified LM (Figure 5c). However, no significant differences relative to the control were found in spontaneous swimming speed (Figure 5d) or phototaxis‐associated vertical swimming velocity (Figure 5e), both of which are sensitive behavioral indicators of neuromuscular function in aquatic organisms [32]. These results suggest that the double‐emulsified LM did not induce detectable acute immobilization toxicity during 48‐h exposure, while causing only minor sublethal effects within this exposure timeframe. However, both spontaneous swimming speed and phototaxis‐associated vertical swimming velocity decreased after prolonged 96‐h exposure (Figure S13), suggesting that longer exposure induces sublethal behavioral effects. Further dose‐ and duration‐dependent toxicity studies, together with the development of strategies to improve the safety profile of LM formulations, will therefore be necessary for applications involving sustained in vivo exposure.

#### 2.4.2 In Vitro Therapeutic Evaluation Using 3D Tumor Spheroid Model

The therapeutic potential of the double‐emulsified LM was assessed using BT‐474 spheroids, which served as a HER2‐positive breast tumor model representative of invasive ductal carcinoma (Figure 5f–k) [33]. A droplet‐based microfluidic system was used to generate uniform spheroids with high reproducibility for subsequent anticancer efficacy evaluation (Figure S14). Owing to their dense cellular architecture and limited mass transport, 3D spheroid models provide a more stringent platform for assessing therapeutic efficacy than conventional two‐dimensional monolayer cultures [34, 35]. The spheroids had diameters of approximately 270 µm, making deep intratumoral penetration of the microscale double‐emulsion droplets (∼12 µm) unlikely. Nevertheless, 24 h after administration of the double emulsion, the optical transmittance of the spheroids decreased markedly compared with the blank control, suggesting effective localization and retention of the double‐emulsified LM near the spheroid surface (Figure S15 and the left column of Figure 5f, denoted as *Before*).

To examine photothermally induced therapeutic activity, spheroids treated with LM/OA/W or the single‐emulsion formulation (LM/W), together with untreated blank spheroids, were exposed to 808 nm NIR irradiation for 10 min. Photothermal measurements conducted under conditions matching those used for spheroid treatment showed equilibrium temperatures of 38.0°C, 41.2°C, and 46.6°C for the blank, LM/W, and LM/OA/W systems, respectively (Figure S16). The highest temperature observed for LM/OA/W indicates its greater photothermal heating capability under the tested conditions. Immediately following irradiation, the normalized area of the LM/OA/W‐treated spheroids decreased to 90.1 ± 2.5% of the initial value, whereas no comparable decrease was observed in the blank or LM/W groups (the middle column of Figure 5f, denoted as *Laser*, and Figure 5h). This rapid decrease in spheroid area is consistent with acute photothermally induced injury [36]. After an additional 24 h incubation, the normalized spheroid areas of the blank and LM/W groups increased to 113.4 ± 1.3% and 105.4 ± 6.1% of their initial values, respectively, whereas that of the LM/OA/W group remained nearly unchanged at 89.3 ± 4.2% (the right column of Figure 5f, denoted as *24 h*, and Figure 5h). Importantly, suppression of spheroid expansion or delayed regrowth can be considered a particularly relevant indicator of therapeutic response in 3D tumor spheroid models, as it reflects the capacity of the residual cells to resume collective growth after treatment [37]. In the matched nonirradiated groups, the spheroid areas increased comparably to above 110% of their initial values over the same incubation period (Figure 5g,h), consistent with continued spheroid growth in the absence of laser exposure. Thus, the absence of post‐treatment spheroid expansion observed specifically for the irradiated LM/OA/W group suggests that the initial photothermal injury limited its subsequent growth capacity [33, 37, 38]. Cell viability was further evaluated after dissociation of the spheroids. In the absence of irradiation, all three groups maintained approximately 100% viability, with no evidence of formulation‐associated cell death during the experimental period (Figure 5j,k). Among the irradiated groups, the blank control retained high cell viability (96.5 ± 1.9%), whereas the LM/W and LM/OA/W groups exhibited reduced viabilities of 86.3 ± 5.5% and 82.9 ± 4.4%, respectively (Figure 5i,k). The reduction observed for the LM/OA/W group was statistically significant relative to the irradiated blank control, as indicated in Figure 5k. Collectively, these results indicate that LM/OA/W induced a measurable, although moderate, laser‐associated cytotoxic response while suppressing post‐treatment spheroid expansion.

To evaluate the contribution of the carrier, an LM‐free OA‐*in*‐water (OA/W) emulsion stabilized with PVA was also tested. However, spheroids exposed to OA/W underwent severe disaggregation during the 24 h preincubation period, before the scheduled laser irradiation. Because the intact 3D spheroid architecture was no longer available at the irradiation time point, the planned comparison between irradiated and nonirradiated OA/W groups could not be meaningfully performed using the same spheroid‐area and morphological endpoints applied to the other groups. The OA/W condition was therefore excluded from the quantitative intact‐spheroid analysis. We note that spheroids treated with nonirradiated LM/OA/W, despite exposure to the same nominal amount of OA, retained their intact 3D architecture, continued to expand, and maintained approximately 100% viability over the same experimental period. These observations indicate a marked formulation‐dependent difference in spheroid responses to the LM‐free OA/W and LM‐containing LM/OA/W emulsions, although the molecular origin of this difference remains unresolved. Accordingly, the biological response of LM/OA/W is interpreted at the formulation level rather than being attributed exclusively to any individual component.

#### 2.4.3 Intermediate Oil Phase as a Versatile Reservoir for Multifunctionality

Finally, we emphasize that the intermediate oil phase of the proposed double emulsion can serve not only as a structural component, but also as a versatile reservoir for additional functional materials. One representative example is the incorporation of therapeutic cargos. Following photothermal therapy, localized delivery of secondary therapeutic agents, such as anti‐inflammatory or antimicrobial molecules [39], may provide additional therapeutic benefits; however, direct encapsulation within the LM phase or complexation on the oxide surface can be challenging for many compounds. In contrast, numerous hydrophobic therapeutic molecules, including curcumin and thymol [40, 41], are readily soluble in slightly polar oils such as OA. Accordingly, the intermediate OA phase can function as a microdomain for the incorporation and retention of such cargos. As demonstrated in Figure 5l, CLSM images reveal selective localization and stable retention of a lipophilic fluorescent dye within the dispersed OA domains.

In addition, the proposed double‐emulsion system can be readily extended to magnetically responsive formulations. Magnetic nanoparticles such as Fe_3_O_4_ can be co‐encapsulated within the OA phase through a mechanism analogous to the OA‐assisted dispersion of LM nanodroplets [42]. As a proof of concept, double‐emulsion droplets containing both LM and Fe_3_O_4_ nanoparticles were successfully prepared (Figure 5m). Upon application of an external magnetic field using a neodymium magnet, the droplets exhibited magnetically guided movement toward the magnetic source in an aqueous medium (Figure 5n and Movie S7). Collectively, these proof‐of‐concept demonstrations suggest that the aqueously stable and photothermally active LM double emulsions developed here may be further adapted, using established droplet‐based loading and transport strategies, to incorporate additional application‐specific functionalities.

## 3 Conclusion

In summary, we demonstrated a simple, batch‐processable oil‐mediated double‐emulsification strategy that improves the aqueous formulation stability of LM and enables durable, localized photothermal heating, together with preliminary biological evaluation. By pre‐dispersing EGaIn nanodroplets in OA as a reactive intermediate oil phase and subsequently emulsifying the resulting dispersion in water, a yolk–shell‐like LM/OA/W double emulsion was formed through wettability‐driven confinement. The intermediate OA phase limited water access to the LM droplets, thereby reducing their transformation into crystalline hydroxide products and preserving their spherical morphology during prolonged aqueous storage and repeated NIR irradiation. Beyond aqueous stabilization, the double‐emulsion architecture spatially retained photothermally active LM near the irradiation region and reduced the convection‐mediated redistribution of generated heat. Consequently, the LM/OA/W formulation exhibited a greater and more durable local temperature rise than the direct LM/W dispersion under identical initial LM loading and irradiation conditions. Preliminary biological studies further demonstrated measurable photothermal suppression of 3D breast cancer spheroid expansion. At the tested dilution, the double‐emulsified LM formulation did not elicit greater acute immobilization toxicity toward *D. magna* than the directly dispersed LM control. Finally, the incorporation of a lipophilic dye and magnetic nanoparticles as proof‐of‐concept additives demonstrated that the intermediate OA phase can also serve as a reservoir for additional functional materials, highlighting the modular nature of the platform. Collectively, this work establishes OA‐mediated double emulsification as a simple and versatile approach for developing aqueous LM‐based photothermal formulations with improved stability, durable local heating, and the potential for further functional integration.

## Funding

This work was supported by a National Research Foundation of Korea (NRF) grant funded by the Ministry of Education (RS‐2025‐25421959, RS‐2025‐25414925) and the Korea government (MSIT) (RS‐2025‐16065277).

## Conflicts of Interest

The authors declare no conflicts of interest.

## Supporting information




**Supporting File 1**: smtd71023‐sup‐0001‐SuppMat.docx.

**Supporting File 2**: smtd71023‐sup‐0002‐MovieS1.mp4.

**Supporting File 3**: smtd71023‐sup‐0003‐MovieS2.mp4.

**Supporting File 4**: smtd71023‐sup‐0004‐MovieS3.mp4.

**Supporting File 5**: smtd71023‐sup‐0005‐MovieS4.mp4.

**Supporting File 6**: smtd71023‐sup‐0006‐MovieS5.mp4.

**Supporting File 7**: smtd71023‐sup‐0007‐MovieS6.mp4.

**Supporting File 8**: smtd71023‐sup‐0008‐MovieS7.mp4.

## Data Availability

The data that support the findings of this study are available from the corresponding author upon reasonable request.
