# Supplementary material for: Oleic Acid‐Mediated Double Emulsification Enables Enhanced Aqueous Stability and Localized Photothermal Heating of Liquid Metal Droplets
Source: Small Methods. 2026 Sep 9;10(19):e71023. doi: 10.1002/smtd.71023 (PMC13646033; doi:10.1002/smtd.71023)
Supplement: Supplementary file 1 [file SMTD-10-e71023-s005.docx]

Supporting Information

Oleic Acid -Mediated Double Emulsification Enables Enhanced Aqueous Stability and Localized Photothermal Heating of Liquid Metal Droplets

*Eunju Na, Youngwon Kim, Jihye Kim, Sang Kug Chung, Yongmin Cho,^*^ Bongseop Kwak,^*^ Joohyung Lee^*^*

E. Na, J. Kim, Y. Cho, J. Lee

Department of Chemical Engineering, Myongji University, Yongin, Korea
E-mail: Y. Cho ([yongmincho@mju.ac.kr](mailto:yongmincho@mju.ac.kr)), J. Lee ([ljbro@mju.ac.kr](mailto:ljbro@mju.ac.kr))

Y. Kim, B. Kwak
College of Medicine, Dongguk University, Goyang, Korea

E-mail: B. Kwak ([bskwak@dongguk.ac.kr](mailto:bskwak@dongguk.ac.kr)),

S. K. Chung

Department of Mechanical Engineering, Myongji University, Yongin, Korea

**Experimental Details**

**Sample preparation.** The LM/OA/W double emulsion was prepared via a two-step sequential emulsification process involving immiscible fluid phases. First, a LM/OA primary emulsion was fabricated by dispersing 0.625 g of EGaIn (75 wt% Ga and 25 wt% In, Suzhou Chuanmao Metal Materials, China) into 5 mL of OA (Daejung Chemicals & Metals, Korea) contained in a 20 mL scintillation vial. The mixture was ultrasonicated using an HD4200 ultrasonic homogenizer (Bandelin, Germany) at 30% amplitude and 20 kHz for 10 min (3 s on / 1 s off). To prevent excessive temperature rise, the process was carried out in an ice bath. For the preparation of the double emulsion, a 1 wt% aqueous PVA solution was first prepared by dissolving poly(vinyl alcohol) (PVA, Daejung Chemicals & Metals, Korea) in deionized water (resistivity: 18.2 MΩ·cm, Direct-Q water purification system, Millipore, USA). For secondary emulsification, 5 mL of the LM/OA primary emulsion was added to 5 mL of the PVA solution and homogenized using an HGA-10 rotor–stator homogenizer (LB Lab Korea, Korea) at 30% speed for 1 min. To prepare double emulsions with varying OA:W volume ratios, the primary emulsion was first prepared identically and then concentrated via centrifugation. The concentrated primary emulsion was subsequently mixed with the PVA aqueous solution to achieve the desired OA:W ratio at a fixed total volume of 10 mL, while maintaining the overall LM concentration at 1 vol%. For control samples, DOD-based emulsions (with and without PVA) were prepared following the identical procedure described above, except that OA was replaced with DOD (Kanto Chemical, Japan). For the preparation of magnetically responsive double emulsions, 0.334 g of Fe_3_O_4_ nanopowder (US Research Nanomaterials, USA) was co-dispersed with 0.666 g of LM in 10 mL of OA during the primary emulsification step, followed by the same ultrasonication and secondary homogenization procedures.

**Characterization of the LM/OA primary emulsion.** The solvodynamic diameter of LM droplets dispersed in the LM/OA primary emulsion was measured by dynamic light scattering (DLS) using a Zetasizer ZS90 (Malvern, UK). The morphology and elemental composition of the resulting LM nanodroplets were examined using transmission electron microscopy equipped with energy-dispersive spectroscopy (TEM-EDS; JEM-2100F, JEOL, Japan). The colloidal stability of the primary emulsion was evaluated using a Turbiscan Lab (Formulaction, France) by monitoring the time-dependent variation in backscattering intensity of 880 nm near-infrared (NIR) light across the sample height.

**Characterization of the LM/OA/W double emulsion.** The yolk–shell structure of LM/OA/W double emulsions with different OA:W ratios was observed using optical microscopy (BX51, Olympus, Japan). To evaluate the reproducibility of the resulting double-emulsion structure, three independent emulsion batches were prepared at an OA-to-water ratio of 5:5, and three aliquots were collected from each batch for optical microscopy analysis. Only OA droplets containing optically identifiable LM were included in the size analysis. Between 129 and 318 droplets were analyzed per image, yielding a total of 1847 droplets across nine optical micrographs. The size distribution, mean diameter, and standard deviation of the analyzed OA droplets were determined separately for each batch. In addition, an apparent two-dimensional confinement fraction, $F_{\mathrm{conf},2D}$, was calculated from six randomly selected optical micrographs acquired at higher magnification as a practical quantitative measure of the extent to which LM was confined within the OA droplets:

$$F_{\mathrm{conf},2D}=\frac{\sum A_{\mathrm{LM},\mathrm{inside}}}{\sum A_{\mathrm{LM},\mathrm{inside}}+\sum A_{\mathrm{LM},\mathrm{outside}}},$$

where $A_{\mathrm{LM},\mathrm{inside}}$ and $A_{\mathrm{LM},\mathrm{outside}}$ denote the projected areas of optically detected LM located inside and outside the OA droplets, respectively. Detailed image analysis protocol, performed with an in-house Python-based annotation tool, is described as follows: Seed masks were generated by detecting OA candidates with a multi-scale Hough circle transform followed by non-maximum suppression and LM candidates by local intensity thresholding followed by connected-component labeling (minimum area, 6 pixels). The seed masks were then manually corrected. Each LM polygon was intersected with the boundary of its enclosing OA, and the fraction of its projected area enclosed within the OA was calculated. LM polygons with at least 90% of their projected area enclosed by the OA boundary were classified as encapsulated (inside); otherwise, they were classified as free (outside). The area terms in the calculation of $F_{\mathrm{conf},2D}$ were summed over all LM polygons in the field of view. The calculated value is an apparent projected-area metric and should not be interpreted as a three-dimensional encapsulation efficiency or a number-based encapsulation fraction. To distinguish the oil and aqueous phases within the double emulsion, confocal laser scanning microscopy (SP8 X, Leica, Germany) was employed. For fluorescence imaging, OA and the aqueous phase were selectively stained with Nile Red (Sigma Aldrich, USA) and fluorescein sodium salt (Sigma Aldrich, USA), respectively. The internal structure of the oil phase (OA phase) containing LM nanodroplets was further analyzed using a cryogenic scanning electron microscopy system (cryo-SEM; Quanta 3D FEG, FEI, Netherlands). Sample preparation followed a previously established protocol for canola oil-based W/O/W emulsions; detailed procedures can be found in the corresponding literature (*ACS Omega*, **2025**, *10*, 28372–28381). To examine morphological changes upon aging, LM microdroplets and their oxidized derivatives extracted from aged double emulsions and control samples were characterized using scanning electron microscopy (SEM; S-4800, Hitachi, Japan). The phase transition behavior of each component in the LM/OA/W double emulsion was analyzed via differential scanning calorimetry (DSC) using a DSC 200 F3 Maia apparatus (Netzsch, Germany). Measurements were conducted under a nitrogen atmosphere during cooling from 30 to −150 °C at a scanning rate of 5 °C/min.

**X-ray analysis.** X-ray photoelectron spectroscopy (XPS) was employed to analyze the surface composition of LM nanodroplets generated in different oil phases. For sample preparation, LM droplets were isolated from LM/OA and LM/DOD primary emulsions by washing with excess hexane. The washing process consisted of dilution with hexane, followed by centrifugation (4000 rpm, 10 min), and removal of the supernatant. The collected droplets were subsequently dried for 24 h under ambient conditions. Surface chemical analysis was conducted using a K-Alpha+ XPS apparatus (Thermo Fisher Scientific, USA) equipped with an Al Kα microfocused monochromator (1486.6 eV). For depth profiling, Ar ion beam etching was performed at an energy of 0.5 keV with a raster size of 1 mm × 1 mm. For X-ray diffraction (XRD) analysis, residues were extracted from double emulsions aged for 7 days (LM/OA/W) and control samples (DOD-based emulsion with PVA) using the same washing procedure described above. The XRD patterns were obtained using an Aeries 600 diffractometer (Malvern Panalytical, Netherlands) over a 2θ range of 5°–90°.

**Wettability test.** A droplet of LM was first introduced into a quartz vessel containing either OA or DOD (as a control) using a pipette. Subsequently, a droplet of deionized water or 1 wt% PVA aqueous solution was gently deposited onto the LM droplet. The wetting behavior was then visually observed.

**Photothermal properties.** Double emulsion (LM/OA/W)~~,~~ and single emulsions (LM/OA and LM/W), all with a fixed LM concentration of 1 vol%, were loaded into 1.5 mL polypropylene microcentrifuge tubes (Neurex, Korea) and vertically mounted using a sample clamp. An 808 nm near-infrared (NIR) laser (LaserTo, Hong Kong; output power: 500 mW, beam diameter <2.5 mm) was irradiated onto the upper region of the sample for 10 s, followed by natural cooling at room temperature. The temperature evolution at the irradiated region was monitored using an infrared camera (Optris Xi 400, Optris Infrared Sensing LLC, USA). For the LM/OA/W samples, seven heating–cooling cycles were performed at one cycle per day over seven days to evaluate repeatability and durability of its photothermal response. To assess structural durability, optical micrographs acquired before irradiation were compared with those obtained after the seven daily photothermal cycles. After the final cycle, the emulsion was washed with hexane according to the same protocol described above, and the recovered LM microdroplets were examined by SEM. To quantitatively analyze the spatial propagation of heat in the LM/OA/W and LM/OA formulations, the infrared thermogram acquired at the time point of maximum temperature during the laser-on period was analyzed on a pixel-by-pixel basis using a custom Python script. At each temperature threshold (ΔT = 10, 20, and 30 °C above baseline), the connected thermal footprint containing the maximum-temperature position was identified. A second-moment equivalent ellipse was then determined from the spatial distribution of pixels within the footprint. The anisotropy index was calculated as the ratio of the major to minor axis lengths of the equivalent ellipse, with a value of 1 indicating an isotropic, circular distribution and larger values indicating increasing anisotropy. To assess the photothermally induced heating of the LM/W and LM/OA/W formulations under conditions matching those used in the spheroid-treatment experiments, each stock formulation (LM content, 1 vol%; OA:W = 1:9 for LM/OA/W) was diluted in culture medium at a culture medium-to-stock formulation ratio of 99:1 (v/v). A blank culture-medium control was prepared in parallel. The samples were then irradiated from above for 10 min using the same NIR irradiation conditions employed for spheroid treatment, while their temperature changes were monitored using the infrared camera.

***Daphnia magna* culture and synchronization.** *Daphnia magna* (*D. magna*) were obtained from the National Institute of Environmental Research (NIER, Korea) and maintained under controlled laboratory conditions compatible with OECD Test Guideline (TG) 202. The organisms were cultured in Elendt M4 medium at 20 °C under a 16 h light/8 h dark photoperiod. The culture medium was replaced every 48 h, and the daphnids were fed daily with the green alga *Chlorella vulgaris* at a concentration of 1 × 10⁵ cells/mL per organism, with the feeding volume adjusted according to the population density. To obtain age-synchronized neonates less than 24 h old, all neonates present in the stock cultures were removed, and newly produced neonates were collected during the subsequent 24 h period and randomly assigned to the control and treatment groups. The experiments involving *D. magna* did not require ethical approval under the regulations of the Republic of Korea. *D. magna* is an invertebrate crustacean and is outside the scope of the Korean Animal Protection Act and the Laboratory Animal Act, which define regulated animals and laboratory animals, respectively, in terms of vertebrate animals. Therefore, institutional animal care and use committee (IACUC) approval and an associated approval number were not applicable to the present study.

***Daphnia magna* exposure and acute immobilization assay.** Acute immobilization assays were conducted using synchronized *D. magna* neonates according to the general procedure of OECD TG 202. A total of 60 neonates were evaluated in each group (n = 60 per group). The test groups included a control group without emulsion exposure, an LM/W single-emulsion group, and an LM/OA/W double-emulsion group (OA:W = 1:9). The stock emulsion contained 1 vol% LM and was diluted 10,000-fold in Elendt M4 medium. The final LM concentration and emulsion dilution ratio were kept identical between the LM/W and LM/OA/W groups. The animals were exposed to the test formulations for 48 h at 20 °C under the same light/dark cycle used for culture. The number of non-immobilized individuals was recorded after 24 and 48 h exposure. Daphnids were considered immobilized when they were unable to swim after gentle agitation of the test vessel. After 48 h exposure, representative animals were imaged using bright-field microscopy to examine the distribution of ingested LM-associated materials within the digestive tract.

**Body size analysis.** Body size was measured after 48 h exposure using bright-field microscopic images. The body length of each *D. magna* individual was defined as the straight-line distance from the top of the head to the base of the tail spine and quantified using ImageJ software. At least 30 individual *D. magna* were evaluated in each group (n ≥ 30 per group), with the exact sample size varying among groups. Data are presented as the mean ± SE. Statistical comparisons were made against the blank control without emulsion exposure. Statistical significance was evaluated using the Kruskal–Wallis test followed by Dunn’s post hoc test with Bonferroni correction (*adjusted p < 0.05, **adjusted p < 0.01, ***adjusted p < 0.001, and ****adjusted p < 0.0001; N.S., not significant).

**Swimming activity and phototaxis-associated behavior.** Spontaneous swimming activity and phototaxis-associated vertical swimming behavior were analyzed after 48 h exposure using a custom-designed observation tank and a machine-learning-based tracking algorithm, as described previously (*Aging Cell* **2022**, *21*, e13571). For swimming analysis, individual *D. magna* were transferred to the observation chamber, and their movement was recorded under controlled imaging conditions. Swimming speed was calculated from the displacement of each animal over time. Phototaxis-associated behavior was evaluated by recording vertical movement under a controlled light stimulus for 20 s. The light source was positioned above the observation chamber to induce vertical phototactic movement. Vertical swimming velocity was calculated along the direction of the light stimulus. Positive and negative values indicate movement toward and away from the light source, respectively. To assess delayed sublethal behavioral effects, an additional cohort was exposed to the same test formulations for 96 h and analyzed using the same behavioral assays. The 96 h behavioral assay was performed as an extended sublethal assessment and was not used as part of the OECD TG 202 acute immobilization endpoint. At least 30 individual *D. magna* were evaluated in each group (n ≥ 30 per group), with the exact sample size varying among groups. Data are presented as the mean ± SE. Statistical comparisons were made against the blank control without emulsion exposure. Statistical significance was evaluated using the Kruskal–Wallis test followed by Dunn’s post hoc test with Bonferroni correction (*adjusted p < 0.05, **adjusted p < 0.01, ***adjusted p < 0.001, and ****adjusted p < 0.0001; N.S., not significant).

**Spheroid fabrication.** BT-474 human breast cancer cells (Korea Cell Line Bank, Seoul, Korea) represent a triple-positive breast cancer subtype expressing estrogen receptor, progesterone receptor, and HER2. Cells were maintained in high glucose DMEM (Gibco, USA) containing 10% fetal bovine serum (FBS, Gibco, USA) and 1% penicillin streptomycin (Gibco, USA). Cultures were passaged every three days or before reaching approximately 80% confluence. BT-474 spheroids were generated using a droplet-based cell encapsulation method. Briefly, cells were suspended in culture medium and introduced into a microfluidic droplet generation system with Novec 7500 engineered fluid (3M, USA) supplemented with 5% FluoSurf C surfactant (FSC 119, MediSphere, Gyeongsangbuk-do, Korea) as the continuous oil phase. Cell containing droplets were collected and incubated at 37 ºC in a humidified atmosphere containing 5% CO_2_ for three days to allow spheroid formation. Mature spheroids were subsequently harvested from the droplets using 1H,1H,2H,2H perfluoro-1-octanol (Sigma Aldrich, USA).

**Evaluation of photothermal anticancer activity.** Fabricated spheroids were individually transferred into an ultra low attachment 96 well plate (CLS3367, Corning, USA), with one spheroid placed in each well. LM/W single-emulsion or LM/OA/W double-emulsion (OA:W = 1:9) containing media were prepared by diluting each stock emulsion (1 vol% LM) in culture medium at a medium to stock emulsion volume ratio of 99:1 (v/v). Each well was supplemented with 200 µL of the prepared medium and incubated at 37 °C in a humidified atmosphere containing 5% CO₂ for 24 h. For photothermal treatment, the samples were irradiated with an 808 nm near infrared (NIR) laser (LaserTo, Hong Kong; output power, 500 mW; beam diameter, <2.5 mm) for 10 min per well, with the laser directed toward the upper region of each sample. The spheroids were then further incubated at 37 °C in a humidified atmosphere containing 5% CO₂ for 24 h. For the nonirradiated controls, the samples subjected to the same 24 h preincubation were incubated for an additional 24 h without NIR irradiation.

**Assessment of anticancer effect.** Spheroid viability was assessed using two complementary staining procedures. First, intact spheroids were stained with Hoechst 33342 and propidium iodide (PI) to visualize the spatial distribution of viable and membrane damaged cells within the spheroid structure. Spheroids were incubated with Hoechst 33342 (Thermo Scientific, USA) and PI (Sigma Aldrich, USA), each diluted at 1:500 in culture medium, for 2 h. Fluorescence images of intact spheroids were acquired using a Leica THUNDER Imager DMI8 (Leica Microsystems, Germany). For quantitative viability analysis, spheroids were dissociated into single cells using 0.25% trypsin EDTA with phenol red (Gibco, USA) for 3 min, followed by gentle pipetting for an additional 3 min. The dissociated cells were then stained with Hoechst 33342 and PI, each diluted at 1:500, for 10 min. Fluorescence images were acquired using the same imaging system, and cell counting was performed using the analyze particles function in ImageJ software (National Institutes of Health, USA). Data are presented as the mean ± SD of four biologically independent experiments per group (n = 4). Statistical comparisons were made against the corresponding non-emulsion-treated blank control under the same irradiation and incubation conditions. Statistical significance was evaluated using the Kruskal–Wallis test followed by Dunn’s post hoc test with Bonferroni correction (*adjusted p < 0.05, **adjusted p < 0.01, ***adjusted p < 0.001, and ****adjusted p < 0.0001; N.S., not significant).

**Tumor size analysis.** The size change ratio, which measures the rate of area decrease or increase, served as a key quantitative metric in this assessment.

$$Relative size change \left( \% \right)= \frac{A}{A_{0}} \times100$$

where A_0_ and A are the initial and final area. Micrographs were acquired using a Leica DMi8 fluorescence microscope (Leica Microsystems, Germany). Spheroid morphology was quantified using an automated segmentation workflow based on Cellpose 2.0 (*Nat. Methods.* **2021**, *18*, 100–106). Bright field images were used to segment the compact spheroid core, which minimized subjective variation in boundary determination and allowed consistent mask generation across experimental groups. The core region was distinguished from peripheral damaged cells and debris by considering bright field morphology and optical contrast. Segmentation masks were then transferred to ImageJ software as regions of interest (ROIs). Projected area and size distribution were measured using automated analysis functions. Data are presented as the mean ± SD of four biologically independent experiments per group (n = 4). Statistical comparisons were made against the corresponding non-emulsion-treated blank control under the same irradiation and incubation conditions. Statistical significance was evaluated using the Kruskal–Wallis test followed by Dunn’s post hoc test with Bonferroni correction (*adjusted p < 0.05, **adjusted p < 0.01, ***adjusted p < 0.001, and ****adjusted p < 0.0001; N.S., not significant).

**Immunofluorescence analysis of tumor spheroids.** Immunofluorescence staining was conducted to characterize the tumor spheroid. Samples were fixed with 4% formaldehyde for 15 min at room temperature and permeabilized with Triton X 100 (Sigma Aldrich, USA) diluted in DPBS for 15 min. Spheroids were incubated with primary antibodies against E-cadherin (1:100, Abcam, UK) and Ki-67 (1:100, Abcam, UK) overnight at 37 °C with gentle shaking. After DPBS washing, appropriate secondary antibodies were applied for 3 h under the same conditions. Nuclei were stained with Hoechst 33342 (1:100), and the samples were washed with DPBS.

**Table S1.** Summary of previously reported strategies for modifying liquid metals.

| **Strategy** | **System** | **Preparation route and complexity** | **Aqueous stability** | **Photothermal response^1^ / irradiation durability** | **Biological evaluation** | **Application** | **Ref.** |
| --- | --- | --- | --- | --- | --- | --- | --- |
| **Inorganic -shell-coated**  **(single emulsion)** | LM@SiO_2_  (~190 nm) | **High**; multistep SiO_2_ coating; multi-hour processing | Long-term aqueous stability not systematically evaluated | Higher $\Delta$T than the SiO_2_-free control under matched conditions / stable over 5 cycles | In vitro & in vivo toxicity | Photothermal therapy | [S1] |
|  | LM@ZrO_2_  (~120 nm) | **High**; multistep ZrO_2_ coating; multi-hour processing | Long-term aqueous stability not systematically evaluated | No direct $\Delta$T comparison with the ZrO_2_-free control  / stable over 5 cycles | In vitro & in vivo toxicity | Photothermal therapy | [S2] |
| **Polymer- or small molecule-coated**  **(single emulsion)** | LM@PVP  (~140 nm) | **Low-to-moderate**; single-step polymer coating; 30 min processing | 30-day aqueous stability demonstrated by SEM | Not evaluated | Not evaluated | Li-ion battery electrodes | [S3] |
|  | LM@POMA  (~170 nm) | **Moderate**;  5 min sonication; requires POMA/toluene emulsion and solvent evaporation | 60-day aqueous stability demonstrated by TEM | Not evaluated | In vitro toxicity | None reported | [S4] |
|  | LM@OA  (~200 nm) | **Moderate**; modified ultrasonic mist-maker-based nebulization; 20 min processing | 120-h aqueous stability demonstrated by TEM | Not evaluated | In vitro toxicity | None reported | [S5] |
| **Double-emulsion-confined** | LM/W/paraffin  (~2.33 mm) | **High**; gravity-induced microfluidic device; controlled multiphase flow | Not applicable (the external phase is paraffin) | Not evaluated | Not evaluated | None reported | [S6] |
|  | LM/PDMS/  ethanol  (~1.91 𝜇m) | **Low**; 8 min processing | Not applicable (the external phase is ethanol) | Not evaluated | Not evaluated | Soft electronics | [S7] |
|  | LM/OA/W  (~12 µm) | **Low**; 11 min processing | 31-day aqueous stability demonstrated by visible inspection /  7-day stability by SEM and XRD | Higher $\Delta$T than the OA-free control under matched conditions / stable over 7 cycles | In vitro & in vivo toxicity | Photothermal therapy | This work |

^1^Absolute temperature-rise and photothermal-conversion-efficiency values were not used for direct interstudy comparison because the LM concentration, sample volume, irradiation wavelength, power density, and irradiation time differed substantially among the studies. Instead, the table indicates whether a matched control comparison and irradiation-cycling durability were demonstrated.

**Figure S1.** (a) Photographs of a representative primary LM/OA emulsion sample monitored for 31 days after preparation and (b) variations in the backscattering intensity (ΔBS) of an 880 nm near-infrared (NIR) beam measured across the sample height (H/H₀: normalized sample height).

**Figure S2.** Observation of the wetting configuration among the three phases: LM (droplet), W (droplet), and OA (bulk).

**Figure S3.** (a) Optical micrographs of three randomly selected aliquots from each of three independently prepared LM/OA/W double emulsions with an OA:W volume ratio of 5:5 (scale bars: 20 μm) and (b) the corresponding size distributions and mean diameters of the LM-containing oil droplets (mean ± SD, n ≥ 129).

**Figure S4.** Optical micrographs and corresponding image-analysis results used to calculate the two-dimensional confinement fraction, $F_{\mathrm{conf},2D}$, of LM within the oil droplets in LM/OA/W double emulsions with an OA:W volume ratio of 5:5.

**Figure S5.** Additional cryo-SEM images of the LM/OA/W double emulsions with an OA:W volume ratio of 5:5 (scale bars: 5 μm).

**Figure S6.** DSC analysis of the LM-free OA/W emulsion containing PVA. The inset shows an enlarged view of the region near −90 °C.

**Figure S7.** Optical micrographs of LM/O/W emulsions with oil phases containing reactive functionalities: (a) canola oil and (b) octanoic acid. The scale bars represent 10 μm. For both emulsion samples, the oil-to-water ratio was fixed at 5:5, and the LM content was fixed at 1 vol% of the total volume. The aqueous phase contained 1 wt% dissolved PVA.

**Figure S8.** One-week stability test in phosphate-buffered saline (PBS) at 37 °C. (a) Photograph of LM/OA/W during storage at 37 °C for one week. The sample contained 1 vol% LM and the W phase consisted of PBS containing 1 wt% PVA; the OA:W volume ratio was 5:5. (b) Optical micrograph of the sample after one week of storage (scale bar: 10 μm). (c) SEM image of LM recovered from the sample after one week of storage (scale bar: 1 μm).

**Figure S9.** Photographs of the LM/OA/W emulsions with various oil-to-water ratios (6:4–1:9): (a1) immediately after preparation and (a2) after 7 days of storage. (b) Optical micrographs of LM/OA/W emulsions prepared at various oil-to-water volume ratios ranging from 6:4 to 1:9 (scale bars: 10 μm). For all emulsion samples, the LM content was fixed at 1 vol% of the total volume, and the aqueous phase contained 1 wt% PVA.

**Figure S10.** (a) Optical micrograph of the LM/OA/W emulsion with an oil-to-water ratio of 1:9, collected from the bottom layer (LM: 1 vol% with respect to the total volume; PVA: 1 wt% with respect to the water phase; scale bar: 10 μm). (b) SEM image of particles collected from the emulsion after 7 days of aging (scale bar: 1 μm).

**Figure S11.** IR thermographs around the irradiation spots of (a) LM/OA and (b) LM/OA/W after 10 s of NIR irradiation. (c) Pixel-resolved analysis of the two thermographs showing the thermal-footprint contours at a ΔT threshold of 20 °C, with the irradiation spot defined as the maximum-temperature point.

**Figure S12.** (a) Photographs showing the macroscopic appearance of the LM/OA sample during seven daily photothermal heating–cooling cycles and (b) the corresponding temperature variations (ΔT) observed over the seven cycles.

**Figure S13.** (a) Spontaneous swimming speed and (b) phototaxis-associated vertical swimming velocity of *D. magna* after 96 h of exposure to diluted LM/W and LM/OA/W emulsions, together with a blank control without emulsion exposure (mean ± SE, n ≥ 30). Positive and negative velocity values indicate movement toward and away from the light source, respectively. Each emulsion-treated group was compared with the blank control using the Kruskal–Wallis test followed by Dunn’s post hoc test with Bonferroni correction (*adjusted *p* < 0.05, **adjusted *p* < 0.01, ***adjusted *p* < 0.001, and ****adjusted *p* < 0.0001; N.S., not significant).

**Figure S14.** Production of spheroids using a droplet-based microfluidic system. (a) The microfluidic droplet generation system was designed with two inlets for surfactant-containing oil and cell suspended culture medium, respectively. The cell suspension was sheared by the continuous oil phase at the flow focusing junction, resulting in the formation of segmented cell-containing droplets that were subsequently collected from the outlet (scale bar: 500 μm). (b) Time-dependent observation of spheroid formation within the droplets revealed that encapsulated cells sedimented to the bottom of each droplet under gravity, where they aggregated and gradually formed compact spheroids (scale bar: 400 μm). (c) Fully formed spheroids were released from the droplets and collected for subsequent experiments (scale bar: 400 μm). (d) Size distribution analysis demonstrated the large-scale production of highly uniform spheroids. (e) Immunofluorescence analysis was performed to assess the maturation and structural integrity of the spheroids. Expression of the proliferation marker Ki-67 and the cell junction marker E-cadherin was confirmed. Immunofluorescence staining visualized these markers: Ki-67 (green), E-cadherin (red), and nuclei (blue) (scale bar: 50 μm).

**Figure S15.** Surface accumulation on spheroids before and 24 h after administration (scale bar: 200 μm). Surface accumulation of the administered material was qualitatively assessed from optical transmittance images. Transmittance heatmaps were generated using the Look Up Tables, LUT, function in ImageJ software.

**Figure S16.** Temperature evolution of blank culture medium (denoted as *Control***)** and culture medium containing LM/W or LM/OA/W at the same formulation dilution used in the spheroid-treatment experiments under identical NIR irradiation conditions

**References for Supporting Information**

[S1] Zhu, P.; Gao, S.; Lin, H.; Lu, X.; Yang, B.; Zhang, L.; Chen, Y.; Shi, J. Inorganic Nanoshell-Stabilized Liquid Metal for Targeted Photonanomedicine in NIR-II Biowindow. *Nano Lett.* **2019**, *19*, 2128−2137.

[S2] Xia, N.; Li, N.; Rao, W.; Yu, J.; Wu, Q.; Tan, L.; Li, H.; Gou, L.; Liang, P.; Li, L.; Meng, X. Multifunctional and Flexible ZrO2-Coated EGaIn Nanoparticles for Photothermal Therapy. *Nanoscale* **2019**, *11*, 10183−10189.

[S3] Liu, Y.; Wang, Q.; Bi, S.; Zhang, W.; Zhou, H.; Jiang, X. Water-Processable Liquid Metal Nanoparticles by Single-Step Polymer Encapsulation. *Nanoscale* **2020**, *12*, 13731–13741.

[S4] Lin, Y.; Genzer, J.; Li, W.; Qiao, R.; Dickey, M. D.; Tang, S.-Y. Sonication-Enabled Rapid Production of Stable Liquid Metal Nanoparticles Grafted with Poly(1-octadecane-*alt*-maleic anhydride) in Aqueous Solutions. *Nanoscale* **2018**, *10*, 19871–19878.

[S5] Tang, S.-Y.; Qiao, R.; Lin, Y.; Li, Y.; Zhao, Q.; Yuan, D.; Yun, G.; Guo, J.; Dickey, M. D.; Huang, T. J.; Davis, T. P.; Kalantar-Zadeh, K.; Li, W. Functional Liquid Metal Nanoparticles Produced by Liquid-Based Nebulization. *Adv. Mater. Technol.* **2019**, *4*, 1800420.

[S6] Fan, Q.; Guo, Y.; Zhao, S.; Bao, B. Generation of Liquid Metal Double Emulsion Droplets Using Gravity-Induced Microfluidics. *RSC Adv.* **2022**, *12*, 20686–20695.

[S7] Liu, S.; Kim S. Y.; Henry, K. E.; Shah, D. S.; Kramer-Bottiglio, K. Printed and Laser-Activated Liquid Metal-Elastomer Conductors Enabled by Ethanol/PDMS/Liquid Metal Double Emulsions. *ACS Appl. Mater. Interfaces* **2021**, *13*, 28729–28736.
